# Strength and Numbers: The Role of Affinity and Avidity in the ‘Quality’ of T Cell Tolerance

**DOI:** 10.3390/cells10061530

**Published:** 2021-06-17

**Authors:** Sébastien This, Stefanie F. Valbon, Marie-Ève Lebel, Heather J. Melichar

**Affiliations:** 1 Centre de Recherche de l’Hôpital Maisonneuve-Rosemont, Montréal, QC H1T 2M4, Canada; sebastien.this@umontreal.ca (S.T.); stefanie.valbon@umontreal.ca (S.F.V.); marieeve.lebel@icloud.com (M.-È.L.); 2 Département de Microbiologie, Immunologie et Infectiologie, Université de Montréal, Montréal, QC H3C 3J7, Canada; 3 Département de Médecine, Université de Montréal, Montréal, QC H3T 1J4, Canada

**Keywords:** T cells, tolerance, T cell receptor signaling, affinity, avidity, anergy, regulatory T cells (Treg), heterogeneity, immunotherapy

## Abstract

The ability of T cells to identify foreign antigens and mount an efficient immune response while limiting activation upon recognition of self and self-associated peptides is critical. Multiple tolerance mechanisms work in concert to prevent the generation and activation of self-reactive T cells. T cell tolerance is tightly regulated, as defects in these processes can lead to devastating disease; a wide variety of autoimmune diseases and, more recently, adverse immune-related events associated with checkpoint blockade immunotherapy have been linked to a breakdown in T cell tolerance. The quantity and quality of antigen receptor signaling depend on a variety of parameters that include T cell receptor affinity and avidity for peptide. Autoreactive T cell fate choices (e.g., deletion, anergy, regulatory T cell development) are highly dependent on the strength of T cell receptor interactions with self-peptide. However, less is known about how differences in the strength of T cell receptor signaling during differentiation influences the ‘function’ and persistence of anergic and regulatory T cell populations. Here, we review the literature on this subject and discuss the clinical implications of how T cell receptor signal strength influences the ‘quality’ of anergic and regulatory T cell populations.

## 1. Introduction

One of the hallmarks of the adaptive immune system is the generation of a large number of antigen receptor specificities, which together allow an individual to recognize any number of pathogens one may encounter throughout their lifetime. The enormous diversity of T cell receptor (TCR) specificities is generated during T cell development in the thymus; recombination of TCR gene segments and the addition of non-templated nucleotides at the recombination junctions is predicted to produce up to 10^15^ unique TCRs [1]. However, not all TCR gene recombination events are functional. Thymocytes ‘test’ their TCR against self-peptide presented by major histocompatibility complex (self-pMHC) molecules expressed on thymic antigen presenting cells (APC) to ensure the generation of an appropriate T cell repertoire. In addition to being functional (able to recognize peptide in the context of MHC), it is important that T cells are not overtly activated by self-peptide in the periphery [2,3]. Thus, numerous mechanisms are in place, in both the thymus and periphery, to prevent the survival or function of autoreactive T cells; these processes are collectively referred to as T cell tolerance.

During T cell development, binding of the αβTCR on immature T cells with low to moderate affinity to self-pMHC molecules on cortical thymic epithelial cells leads to positive selection and results in thymocyte differentiation into mature conventional CD4^+^ or CD8^+^ T cells [2,3]. In contrast, high affinity TCR interactions with self-pMHC presented by dendritic cells (DC), medullary thymic epithelial cells, and B cells can lead to thymocyte death. This process, termed ‘negative selection’, is the primary mechanism of central tolerance, which prevents the egress of autoreactive T cells from the thymus. However, it has been suggested that negative selection is only 60–70% efficient, a level which may have been evolutionarily selected [4,5,6,7]. Indeed, it is possible that the elimination of all self-reactive T cells could lead to a decrease in the diversity of the TCR repertoire that would allow some pathogens to go undetected [8,9,10]. The survival of some self-reactive T cells in the periphery is thought to be important to allow for maximal repertoire diversity; T cells with relatively lower levels of reactivity for self-peptides might respond with high affinity/avidity to non-self-peptides and thus provide a wide range of protection [11]. Other mechanisms of central tolerance include the divergence of autoreactive T cells toward immunoregulatory lineages and, potentially, the induction of an unresponsive state in thymocytes with high affinity for self-pMHC. Nevertheless, some autoreactive T cells inevitably ‘escape’ the thymus and seed the periphery. As such, a second layer of protection against harmful autoreactive T cells, known as peripheral tolerance, is essential to prevent autoimmunity. Similar to the thymus, different processes such as deletion, cellular fate diversion into immunoregulatory lineages, and the induction of an unresponsive state occur in the peripheral lymphoid organs and aid in restricting T cell responses to self-antigen [12].

The strength of TCR interactions with self-pMHC has been shown to play an important role in T cell fate decisions in both the thymus and periphery [2,3]. Even among mature conventional T cell subsets that were long considered homogenous, the extent of their self-reactivity contributes to biases in functional potential [13,14,15,16]. For example, among conventional CD4^+^ αβ T cell populations, a broad range of TCR affinities for self-pMHC support positive selection [2,17]. This leads to significant variation in the level of basal ‘self-reactivity’ between individual cells within the mature T cell population, which influences their response during antigen challenge [18]. CD4^+^ T cells with relatively higher basal self-reactivity predominate during acute antigen challenge and are preferentially recruited into the memory T cell pool [14,19,20]. In addition, there is evidence of T helper biases among CD4^+^ T cells with relatively high versus low affinity for self-peptide [19,20,21,22]. While some of these characteristics might be continually influenced by tonic signals (interactions between naïve T cells and self-pMHC that are necessary for their survival) in secondary lymphoid organs, there is evidence that some functional biases might be imprinted during their differentiation into mature T cells in the thymus based on the strength of positive selection signals [23].

Although many studies have highlighted important roles for the strength of TCR interactions with self-pMHC in the generation of functional heterogeneity among conventional T cell populations, less is known about how differences in TCR signal strength during the induction of tolerance might also contribute to the heterogeneity observed within tolerant T cell populations. Different cell-intrinsic mechanisms prevent overt T cell activation to self- (or other self-associated innocuous) peptides in healthy individuals. These can be broadly divided into deletion, ignorance, anergy, and regulatory T (Treg) cell induction [12]. Deletion occurs when T cells die via apoptosis following high-affinity recognition of self-peptide under non-inflammatory conditions. Of autoreactive T cells that survive, some exist in a state of ‘ignorance’ due to low levels of self-peptide presentation; these cells remain naïve and functional. Other self-reactive T cells that do engage self-pMHC with considerable affinity can become anergic, a term that is used to denote T cells possessing a dysfunctional or hyporesponsive state. Diversion toward the Treg lineage prevents overt autoreactive T cell activation; in addition, these cells possess the ability to regulate or suppress additional inappropriate immune responses [24].

It is becoming increasingly clear that anergic and Treg cells are not homogeneous populations, and new evidence points to the possibility that the strength of TCR signals during their differentiation might imprint individual cells among these populations with distinct ‘functions’ [25,26,27,28,29,30]. Notably, the biology of anergic T cells and Tregs has been extensively studied and excellent reviews exist on these topics [25,31,32]. Here, we focus on how the strength of TCR signals impacts their differentiation into these tolerant states as well as their persistence, reversibility, and function. Further understanding of how the quality and quantity of TCR interactions with self-peptide influence the mode and durability of T cell tolerance has important clinical implications.

## 2. TCR Signal Strength: Affinity, Avidity, and Functional Avidity

**Figure 1:**
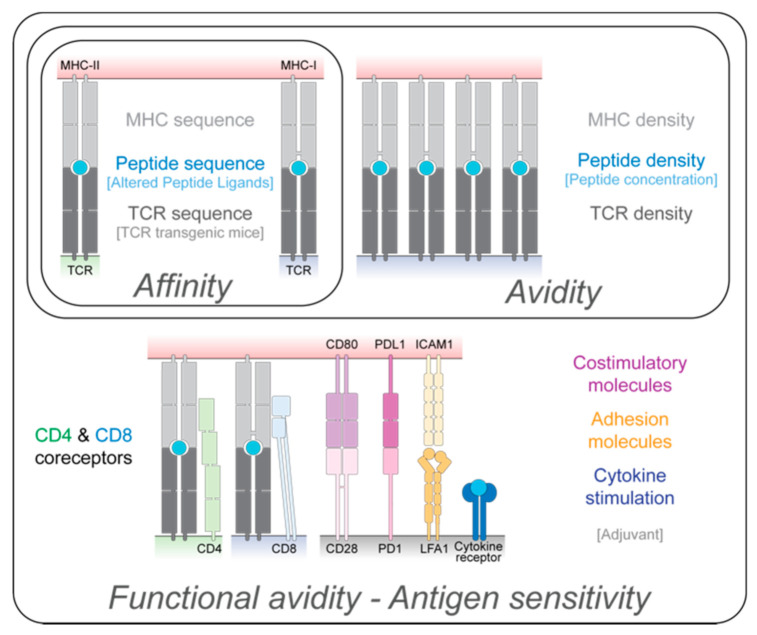
Factors that influence the affinity, avidity, and functional avidity of T cells. T cell receptor (TCR) affinity is defined by the strength of an interaction between a single TCR and a given peptide presented by a major histocompatibility complex (MHC) molecule. It depends on the amino acid sequence of the TCR, the sequence of the peptide being presented, and the MHC allele. TCR avidity is dependent on both the affinity of a single TCR molecule for a given pMHC and the number of TCR-pMHC engagements. Functional avidity, also known as antigen sensitivity, considers also the influence of co-receptors, co-signaling molecules, adhesion molecules, and cytokines that might influence the overall intensity and duration of the TCR signal. Multiple approaches (in brackets) can be used to manipulate the different parameters to influence TCR signal strength.

TCR signal strength is a critical component of T cell fate determination. Different parameters underlie TCR signal strength and can be broadly divided into affinity, avidity, and functional avidity [33] (Figure 1). First, TCR affinity is characterized by the strength of an interaction between a single TCR molecule and a given peptide presented by MHC. It is the result of three critical parameters: the amino acid sequence of the TCR, the sequence of the peptide being presented, and the MHC allele itself (e.g., the I-Ag7 murine MHC complex is a poor peptide binder [34]). Second, TCR avidity is a result of the combined strength of the interactions between multiple TCR and pMHC molecules; it is, therefore, dependent on both the affinity of a single TCR molecule for a given pMHC and the number/density of TCR-pMHC engagements. The latter is, thus, strongly dependent on the concentration of the antigen being processed and presented. Lastly, functional avidity, also known as antigen sensitivity, considers not only TCR avidity, but also the influence of signaling by different co-receptors, positive/negative co-stimulatory molecules, cytokines, and adhesion molecules that might influence the overall intensity and duration of the TCR signal. Thus, functional avidity describes how well a T cell can respond to different concentrations of available peptides, is finely tuned by a wide range of parameters (TCR affinity, TCR and pMHC concentration, co-stimulation, cytokine milieu, etc.), and strongly influences the breadth and quality of a T cell response (Figure 1). Although the affinity of the TCR to a given peptide remains the same during the lifetime of a T cell (unlike their B cell counterparts, there is no somatic mutation of the TCR sequence), avidity and functional avidity are known to change throughout an immune response [35,36,37].

Each of the parameters mentioned above can impact TCR signaling through the activation of different intracellular pathways, the induction of distinct gene expression profiles, and thus, ultimately influence T cell fate and function. Following TCR-pMHC interaction, Lck, a tyrosine kinase associated with the intracellular domain of the CD4 and CD8 co-receptors, initiates the phosphorylation of the immunoreceptor tyrosine-based activation motifs of the CD3 subunit. These phosphorylated residues create docking sites for the recruitment and activation of the kinase ZAP70 which, in turn, phosphorylates LAT allowing for the recruitment of adaptor proteins. This chain of events leads to the formation of a multimolecular LAT signalosome resulting in cytoskeletal reorganization, an increase in intracellular calcium, and the activation of proteins such as MAP kinases, PKC, and calcineurin. This process results in changes in gene expression due to the activation of transcription factors such as AP-1, NF- κB, and NFAT [38]. It is important to note that the strength of TCR signal perceived by a T cell influences multiple steps of this pathway. For example, differences in TCR-pMHC affinity can lead to the phosphorylation of specific serine residues of Lck which, in turn, can recruit or block phosphatases that negatively regulate TCR signaling to impact gene activation [39]. An elegant study has also shown that subtle changes in TCR-pMHC affinity can impact the rate of calcium flux as well as the phosphorylation of LAT and ERK proteins [40]. While the regulation of TCR signaling at different stages is beyond the scope of this review (and has been well reviewed elsewhere [38,41]), it is important to consider that differences in TCR signals (due to changes in affinity, avidity, or functional avidity) can be distinguished by T cells based on changes in the activation of intracellular pathways ultimately impacting T cell outcomes. The relative strength of TCR signals involved in the induction of anergy or Treg induction and their potential link to tolerant phenotype are highlighted further in the subsequent sections.

There are multiple approaches by which one can modulate affinity, avidity, and functional avidity to experimentally test the influence of each component of TCR signal strength on T cell fate choice and function (Figure 1). To analyze the effect of affinity on T cell biology, many studies have taken advantage of altered peptide ligands (APL), a set of peptides in which specific amino acids are changed that lead to a decrease or increase in affinity relative to a known TCR-pMHC interaction [42]. Transgenic (Tg) T cells possess different peptide binding sequences while maintaining specificity to the same cognate peptide, thus, different affinity (e.g., OT-I vs. OT-3) are also routinely used to analyze the effect of TCR affinity to a known cognate peptide (i.e., Ovalbumin (OVA)) [43]; however, one must consider any differences in cell surface levels of TCR expression or other immunoregulatory molecules in these models, which may also change TCR avidity. One way to alter TCR avidity is by changing the amount of the available peptide; increasing peptide concentration can lead to stronger TCR signaling, while the same peptide at a lower concentration induces reduced TCR signaling. Lastly, modification to the environmental milieu (adjuvant, cytokine supplementation, etc.) can be used to fine-tune antigen sensitivity. While some groups have managed to establish models to dissect the specific role of pMHC-potency (affinity) versus that of pMHC density (avidity), comparing the T cell response to high concentrations of low affinity peptide to that of low concentrations of high affinity peptide [44], it is often difficult to distinguish the influence of affinity, avidity, and functional avidity on T cell fate choices. In addition, this nomenclature is often used interchangeably or incorrectly [33,45]. In this review, we explore the effect of these components, while noting the experimental caveats, in dictating the ‘quality’ of T cell tolerance.

## 3. T Cell Anergy

The immune system implements different layers of regulation to prevent immune pathologies such as the establishment of a functionally unresponsive state in autoreactive T cells that is known as anergy. The word anergy is broadly used to characterize cells in a dysfunctional state, which are unable to respond properly when triggered by an activating stimulus [25]. Although some groups have suggested that T cells can be released from the thymus in this state (central tolerance) [46,47], most studies have reported anergy induction in mature T cells in the periphery (peripheral tolerance) [12].

Anergy has been broadly, although not perfectly, divided into two categories. The first category is referred to as clonal anergy; these cells do not proliferate but are still able to produce effector cytokines, to a certain degree, following subsequent TCR stimulation. Antigen is not required to maintain this dysfunctional state though clonal anergy may be reversible by the addition of IL-2. Adaptive tolerance, on the other hand, is characterized by the suppression of both cell proliferation and cytokine production. Adaptive tolerance is not reversed by the addition of IL-2, and continued antigen exposure appears to be required for the maintenance of this phenotype [25]. Although these two anergic states have been fairly well characterized, the nomenclature is not universally applied. Thus, many studies broadly use the word anergy to describe any dysfunction observed in T cells characterized by lack of proliferation, cytokine production, or even a decrease in the phosphorylation of TCR signal intermediates following subsequent stimulation under conditions that would typically activate a naïve T cell.

To date, many studies have shown that the activation of intracellular pathways triggered by the interaction of the TCR with pMHC is crucial for the establishment of this state [48]; however, whether differences in TCR signal strength during the induction of anergy accounts for the heterogeneity in terms of the ‘quality’ or persistence of this dysfunctional state is yet to be fully appreciated. Here we explore the studies that have analyzed how TCR signal strength (following changes in affinity, avidity, or functional avidity) might support this hypothesis.

As a prelude to our discussion, a few additional points must be taken into consideration when reviewing the relevant literature to consider anergy induction and deletion of a T cell population. Analysis of TCR Tg cells specific for a self- or self-associated antigen can be tracked via congenic markers, fluorescent labels (genetic or dyes), antibodies specific to the transgenic TCR chains, and/or tetramers. Tracking autoreactive T cells among a polyclonal population is largely limited to the use of tetramers. Nevertheless, tetramers (or even multimers such as dextramers) are limited in their sensitivity [49]. This limits their effectiveness in detecting T cells bearing antigens with low affinity for a given pMHC. In addition, the TCR complex is downregulated following ‘activation’ [50], although this may not be the case in tolerant conditions [51]. Thus, a decrease in the number of tetramer or anti-TCR stained cells does not always indicate that deletion has occurred and whether anergic T cells are present. Moreover, when focusing on the number of antigen-specific T cell numbers, one should also note the organ(s) being analyzed; a decrease in antigen-specific T cells in lymphoid organs might not correlate with deletion but rather migration to peptide expressing organs (e.g., pancreas, liver, etc.).

### 3.1. CD8^+^ T Cell Anergy

#### 3.1.1. CD8^+^ T Cells Bearing Antigen Receptors with Affinity for Self-pMHC near the Negative Selection Threshold Are Tolerant at Steady-State but Can Induce Autoimmune Pathology

**Figure 2:**
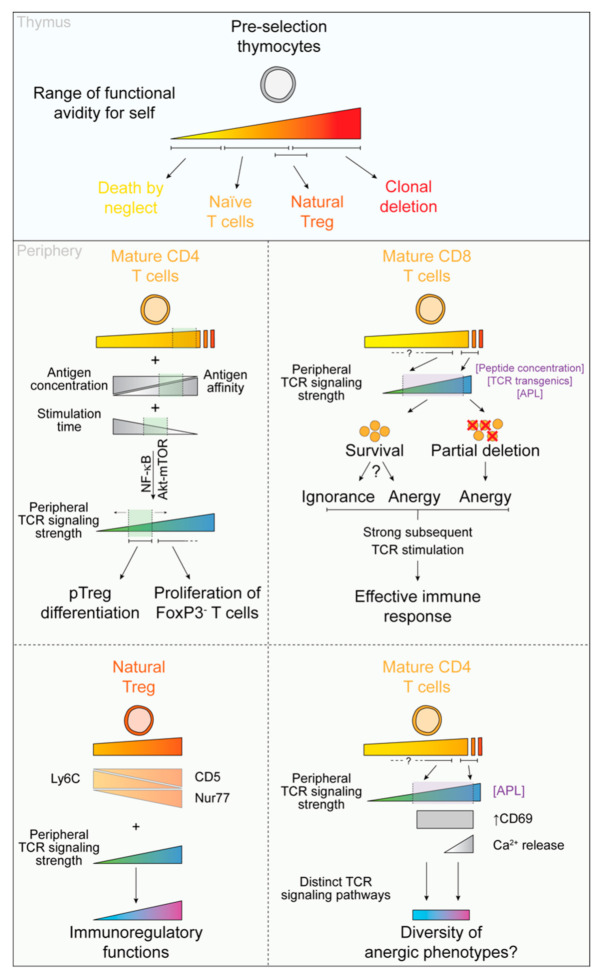
T cell receptor signal strength influences the ‘quality’ of Treg and anergic cells. (Top panel) During T cell development, αβ-lineage thymocytes test the reactivity of their T cell receptor (TCR) to self-peptides. The fate of the developing T cell is dependent on the strength of this interaction. T cells experiencing very low and very high functional avidity to self-peptides are deleted by neglect or clonal deletion, respectively. Low to moderate affinity interactions support the development of conventional CD4^+^ and CD8^+^ T cells. Moderate to high affinity interactions drive regulatory T cell (natural Treg) generation; the strength of these interactions overlap with MHC class II-restricted TCR signals that support both positive and negative selection [69] and are likely influenced by environmental cues that determine cell fate choice in this range of avidity [70]. (Middle and bottom panels) Influence of TCR signals on nTreg function as well as CD4^+^ and CD8^+^ T cell anergy induction and peripheral Treg (pTreg) differentiation.

It has been proposed that CD8^+^ T cells with relatively low affinity/avidity for self-peptide can cause immune pathologies [52], suggesting that mechanisms ensuring tolerance of T cells with a broad range of self-reactivity have been evolutionary selected to prevent the development of such pathologies. However, the relative affinity/avidity of those T cells that can potentially cause harm in terms of the polyclonal population are not clear, and the mechanisms that hold these cells in check are still to be fully defined (Figure 2). A study using CD8^+^ TCR Tg T cells bearing the OT-3 TCR specific to OVA with ‘moderate’ affinity (the affinity of the OT-3 TCR for OVA appears to lie just at the threshold for negative selection), analyzed the impact of TCR self-reactivity on the establishment of peripheral tolerance [43]. This study showed that, in OVA-expressing mice, the percentage of OT-3 Tg cells was reduced by half in the peripheral lymphoid organs as compared to their non-OVA expressing control counterparts. In this model, neither central nor peripheral tolerance led to the complete removal of all self-reactive OT-3 CD8^+^ T cells from the mature T cell repertoire. Nevertheless, OT-3 Tg cells did not attack OVA expressing cells in the periphery, suggesting that peripheral tolerance mechanisms act to prevent activation of T cells, which would ultimately lead to immune pathologies. This tolerant state was broken following infection with a pathogen expressing the cognate antigen, and the activated T cells caused autoimmune pathology [43]. These results support the notion that a significant number of T cells that are not deleted by tolerance mechanisms are generally unable to cause harm at steady-state due to their inability to respond to peripheral levels of cognate peptide in the absence of inflammation.

In complementary models, analysis of mice expressing the OT-I TCR (high affinity for the cognate OVA antigen) as well as OVA or lower affinity APLs suggest that cells possessing an antigen receptor with affinity for the model antigen that lies just above the negative selection threshold in the thymus might present the most harm as they are less efficiently deleted by negative selection as opposed to higher affinity T cells [53]. The antigen-specific T cells that escaped negative selection were tolerant to peripherally expressed antigen at steady-state. However, immunization in the presence of lipopolysaccharide was able to induce diabetes in mice with TCR-pMHC interactions just above the negative selection threshold but not in those with higher affinity T cells. Thus, the tolerant state of these T cells that are just above or below the negative selection threshold are reversible with inflammation whereas the few cells that survive negative selection and are of higher affinity have more stable tolerant phenotypes. Overall, analyses of TCR transgenic CD8^+^ T cells (OT-I vs OT-3) [43], a polyclonal population of CD8^+^ T cells with heterogeneous avidity for OVA [52], or an endogenous polyclonal T cell population in OVA expressing mice [54], suggest that subtle shifts in TCR signal thresholds involved in negative selection may cause significant autoimmune pathology.

#### 3.1.2. Strong TCR Signals Can Lead to CD8^+^ T Cell Anergy or Deletion

Whether the surviving T cells in the models described above are ignorant to the level of physiological peptide present in the periphery or possess a dysfunctional state that can be reversed following strong TCR signaling during infection is not explicitly tested. However, other studies propose that anergy can be induced in CD8^+^ T cells following high affinity/avidity interactions with self-peptide under tolerant conditions [55]. CD8^+^ T cells can acquire an anergic phenotype after exposure to either high affinity APLs or high doses of peptide (high avidity). This dysfunctional phenotype was characterized by a decrease in phosphorylation of TCR signaling intermediates in CD8^+^ T cells; unfortunately, the ability of these cells to produce cytokines was not analyzed [56,57]. The authors proposed that high avidity TCR signaling leads to the deletion of some antigen-specific CD8^+^ T cells while promoting anergy in the remaining cells. The induction of a hyporesponsive state in a portion of the cells prevents the complete deletion of the high avidity self-reactive T cell population, which allows for these cells to be activated and provide protection (by reversing the anergic state) if necessary [56]. Although the precise mechanism of how this might occur is yet to be examined, high avidity CD8^+^ T cells depend on NDFIP1 (a HECT-type ubiquitin ligase activator) to become anergic. NDFIP1-deficient OT-I TCR Tg T cells fail to become dysfunctional under tolerizing conditions with high antigen dose, causing autoimmune pathology in OVA-expressing mice [58]. In addition, it was shown that high affinity mature OT-I CD8^+^ T cells acquire an anergic phenotype when transferred into OVA-expressing mice. These cells possessed lower cytokine production capabilities following ex vivo stimulation with OVA and expressed high levels of PD-1 [27,59]. This phenotype could be reversed following infection with a pathogen expressing the cognate peptide, again suggesting that high peptide levels together with inflammatory cues (leading to a high TCR signaling strength) allow a reversal of the anergic state of CD8^+^ T cells such that they are able to proliferate. Serial stimulation during tolerance reversal leads to an upregulation of the TCR on OT-I cells, which may result in an increase in antigen sensitivity [60]. Lastly, it is important to note that, in contrast to recently activated CD8^+^ T cells, anergic CD8^+^ T cells induced in these conditions are unable to respond to PD-1 (or other checkpoint molecules) blocking therapy, until the anergic state is reversed by strong TCR signaling [59].

Together, these studies suggest that the level of TCR signaling impacts the outcome of self-reactive T cells. Strong TCR signals (due to high affinity/avidity interactions with self-peptide) may lead to either deletion or anergy induction in CD8^+^ T cells. What is clear is that central and peripheral tolerance mechanisms do not remove all potentially harmful T cells from the mature CD8^+^ T cell repertoire [43,52]; yet, these cells do not overtly respond to self-antigen at steady-state, either due to ignorance or to an induced dysfunctional state. In addition, high TCR signaling strength in the presence of inflammation seems to reverse this phenotype allowing for protection against foreign invaders (at the cost of potential collateral tissue damage) and ensuring maximal receptor diversity [43,52,53,54]. Nevertheless, to what extent the initial TCR signaling that dictates CD8^+^ T cell anergy might influence the functionality of these cells, in terms of the quality and/or the persistence of CD8^+^ T cell anergy, is yet to be determined.

### 3.2. CD4^+^ T Cell Anergy

#### 3.2.1. A Spectrum of TCR Signals Can Induce CD4^+^ T Cell Anergy

The relative strength of the TCR signals that induce anergy in CD4^+^ T cells is still being debated (Figure 2). Several groups have suggested that ‘low’ affinity TCR interactions with pMHC are sufficient to induce functional unresponsiveness in this population. For example, low avidity TCR interactions with pMHC can induce anergy if peptide is presented by fully competent APCs (i.e., in the presence of co-stimulation) [61]. The resulting CD4^+^ T cells were unable to proliferate or produce IL-2 in response to subsequent stimulation under otherwise activating conditions. However, this phenotype was sustained for only a short period of time (seven days). Separately, by modulating the time of interaction between TCR-pMHC complexes, another study showed that short TCR-pMHC interactions (i.e., low TCR signaling) can also lead to CD4^+^ T cell anergy. Using peptides with the same TCR residue sequence but different MHC class II anchor residues, it was shown that short-lived T cell-APC interactions can lead to CD4^+^ T cell anergy. This phenotype could also be achieved by low concentrations of long-lived peptides [62]. Yet, other studies suggest that it is relatively higher affinity T cell clones in which anergy is induced and that, in fact, the ‘reversal’ of a tolerant phenotype in a polyclonal population might be due to activation of lower affinity clones that were not initially tolerized [63]. Clearly, more extensive study is needed in order to determine the relative levels of self-reactivity that lead to an anergic phenotype in CD4^+^ T cells. That said, it has been suggested that CD4^+^ T cells with relatively high levels of self-reactivity (i.e., those at the boundary between positive and negative selection) display some characteristics of anergic T cells in that they express markers associated with anergy and are hyporesponsive to stimulation [64].

#### 3.2.2. CD4^+^ T Cell Anergy Is Induced through Distinct Mechanisms Based on TCR Signal Strength

Elegant studies of TCR signaling during CD4^+^ T cell activation and anergy induction have described the dynamic interaction between DC and CD4^+^ T cells as well as the TCR signaling pathways induced downstream of TCR-pMHC interactions of different affinity. Using an MHC class II-restricted TCR Tg T cell population in conjunction with APLs, it was shown that relatively low-, moderate-, and high- affinity peptides can induce CD4^+^ T cell anergy as characterized by a lack of proliferation and decreased IFNγ production [65]. In all three conditions, cells upregulated CD69, an ‘activation’ marker and a molecule important for T cell lymph node retention. However, the affinity of the TCR-pMHC interaction (and, thus, the strength of TCR signaling) drove different signals downstream of the antigen receptor in the absence of inflammation. Stable interactions between CD4^+^ T cells and DCs have been reported under both activating and tolerizing conditions. However, Ca^2+^ flux and T cell deceleration occurred only following the recognition with high potency pMHC complexes, but not in the presence of low- and moderate-affinity interactions, during the induction of anergy in the absence of inflammation [65]. It has been shown that increases in intracellular Ca^2+^ levels are necessary and sufficient for T cell arrest [66,67]. The establishment of an anergic phenotype in the presence of low- and moderate- affinity APLs without substantial Ca^2+^ flux and T cell arrest suggests the formation of an immunological synapse might not be required for tolerance induction [65]. Thus, in tolerizing conditions, the length of interaction between T cells and DCs, TCR signaling pathways, and intracellular Ca^2+^ levels depend on the affinity of the TCR-pMHC interaction. Ultimately, the Ca^2+^-independent induction of anergy by low- and moderate-affinity peptides likely leads to a unique transcriptional program and distinct subset of dysfunctional cells as compared to those cells rendered unresponsive in the presence of high-affinity antigen. Nevertheless, whether the differences in the TCR signaling following exposure to different affinity peptides influence the phenotype of the anergic CD4^+^ T cells has yet to be directly studied.

### 3.3. T Cell Signal Strength and Anergy: Considerations and Open Questions

Overall, the current state of the field prevents us from establishing a complete model of the influence of TCR signaling strength on anergy induction and anergic phenotype. However, significant evidence points to the idea that, for both CD4^+^ and CD8^+^ T cells, high functional avidity interactions can lead to a dysfunctional state or the clonal deletion of autoreactive T cells. T cells with relatively lower avidity TCR-pMHC interaction to self-peptide also can become functionally unresponsive, but where these sit on the self-reactivity ‘spectrum’ of the polyclonal population is less clear (Figure 2).

Anergic T cells appear to be a heterogeneous population in terms of their ability to produce cytokines after subsequent stimulations and the persistence of their phenotype [25]. Although we might infer that the observed differences in TCR signals downstream of relatively lower or higher affinity TCR-pMHC interacts that induce anergy might influence the phenotype of these cells, this has not been studied directly. In addition, direct comparisons of results from different studies are difficult, particularly when considering the impact of ‘low’ and ‘high’ affinity/avidity TCR interactions on T cell anergy. Differences in the approaches to drive anergy induction and the different TCR-pMHC combinations used often preclude direct comparisons of TCR affinity/avidity for peptide.

It is interesting to consider how the mechanism of anergy interacts with other peripheral tolerance mechanisms, in particular, with Treg induction. As we detail in the next section, a similar range of naïve T cell TCR functional avidity may also be optimal for the induction of Treg in the periphery. It is thus tempting to also speculate how autoreactive CD4^+^ T cells are oriented towards anergy versus the Treg cell fate. While cytokines and environmental cues are likely to strongly influence this choice, it is also possible that a distinct range of functional avidity dictates the anergy versus Treg fate choice. To date, this particular question has not been investigated. However, some studies suggest that anergy and Treg induction are not necessarily independent cell fates. Several studies demonstrate that upon reversal of the anergic state, CD4^+^ T cells are able to acquire regulatory functions, express immunomodulatory molecules, and differentiate into Foxp3-expressing cells (reviewed in [68]). While we do not investigate this particular aspect of peripheral tolerance, it is important to note that these tolerant T cell phenotypes are not necessarily independent. In the next sections, we investigate how the diversity of Treg-mediated tolerance, both at the level of their differentiation and their immunomodulatory functions is dictated by the strength of TCR stimulation both in the thymus and in the periphery.

## 4. Regulatory T Cells

Treg can be categorized into two major subsets. A fraction of thymocytes with moderate to high levels of functional avidity to self-peptides differentiates into natural Treg (nTreg). Peripherally induced Treg, referred to as peripheral Treg (pTreg) or induced Treg (although this nomenclature is also often used for in vitro differentiated Treg), arise from mature naïve T cells following recognition of self-peptides [71]. It is important to note that, in the context of pTreg induction, and in particular pTreg induction at the epithelial surfaces, the notion of self is slightly skewed to include innocuous non-self or self-associated antigens. These antigens, often derived from dietary and environmental antigens as well as the microbiota, are present in large quantities at the epithelial interface. Mechanisms, among which pTreg induction is critical, allow for the induction of a tolerant state to these self-associated molecules, hence preventing unwanted inflammation at the interface surfaces. TCR signaling is critically required for the differentiation and function of these immunoregulatory T cell subsets [70], and the strength of the TCR signal during ‘activation’ of Tregs may influence their function. However, how the strength of TCR signals that accompany Treg development in both the thymus and periphery impacts their effector function has only recently emerged (Figure 2).

### 4.1. The Avidity of TCR for Self-Peptide Determines nTreg Fate and Function

#### 4.1.1. Thymic nTreg Fate Decisions and Heterogeneity

Treg induction in the thymus is often referred to as agonist selection. As such, it is suggested that CD4^+^ thymocytes with high affinity to self-pMHC are (negatively selected or) diverted to the Treg lineage as a mechanism of central tolerance. Indeed, several studies suggest that, in addition to cytokine and co-stimulatory requirements, increased TCR affinity and/or avidity for self-peptide preferentially induces nTreg cell development in the thymus over conventional T cell differentiation. As evidence of this, T cells engineered to express TCRs cloned from Treg undergo more robust homeostatic expansion in a lymphopenic environment as compared to T cells expressing antigen receptors derived from conventional CD4^+^ T cells [72]. Additionally, it is suggested that the efficiency of Treg selection, in a setting with a fixed amount of a single model self-antigen, directly correlates with the affinity of the TCR for its ligand [73], although it has been suggested that relatively lower affinity ‘self-antigens’ can support Treg development as compared to higher affinity peptides at higher doses. There is also evidence that avidity, as altered by peptide dose/density of the peptide–MHC complexes presented, also regulates Treg development [74,75,76,77,78]. However, there is a rather broad range of TCR signals perceived by developing CD4^+^ T cells in response to self-pMHC that support Treg differentiation [73]. In fact, the strength of TCR signals associated with Treg development overlaps with MHC class II-restricted thymocytes that undergo both positive and negative selection [69]. The strength of TCR signals that accompany the selection of these cells further directs differentiation of mature Treg cells through distinct progenitor populations; stronger TCR signals support Treg cell differentiation via a CD25^+^ progenitor cell intermediate whereas lower TCR signals are associated with the development of a Foxp3^lo^ Treg progenitor population [29]. The differences in the quality of TCR signals that accompany their development induce Foxp3 via distinct mechanisms and, overall, lead to differences in their transcriptome profiles [29].

#### 4.1.2. Self-Reactivity Instructs nTreg Function 

In addition to determining the nTreg fate, the avidity of nTreg to self-peptide strongly influences their function in the periphery (Figure 2). For example, nTreg-derived from CD25^+^CD4^+^ progenitor cells that are reported to interact with self-antigens with relatively high affinity suppress pathogenic T cells in a mouse model of multiple sclerosis whereas their Foxp3^lo^ progenitor-derived counterparts with relatively lower self-reactivity do not. In addition, levels of CD5, Nur77, and Ly6C used to quantify the relative self-reactivity of thymic and peripheral T cells also parse differences in nTreg function. CD5 and Nur77 levels are directly proportional to TCR self-reactivity whereas bimodal expression of Ly6C on CD4^+^ lineage T cells is inversely associated with the strength of TCR interactions with self-pMHC [69,79,80]. Higher levels of CD5 or lower levels of Ly6C on nTreg correlate with elevated expression of effector molecules and higher suppressive activity both in vitro and in vivo [30,81,82]. In addition, enrichment of Treg populations with different levels of Nur77 expression demonstrated that Nur77^hi^ Treg have a transcriptional signature reflecting a more activated phenotype as compared to their Nur77^lo^ counterparts [83]. Finally, based on the expression of the effector molecules GITR, PD1 and CD25, Triple^hi^ and Triple^lo^ Treg were identified [28]. The authors elegantly show that Triple^hi^ Treg display higher self-reactivity, as evidenced by relatively higher levels of CD5 and Nur77 and have stronger suppressive functions as measured by their ability to suppress lymphoproliferation and T cell-induced colitis than their Triple^lo^ Treg counterparts.

Using a series of Tg mice bearing TCR for an endogenous peptide further demonstrates that TCR affinity for self contributes to the diversity of Treg functional heterogeneity (to note, whether these are thymically or peripherally derived Tregs was not distinguished). Mice expressing eight different MHC class II-restricted TCRs specific to Ins_B9-23_ allowed assessment of the role of TCR self-reactivity on Treg function in the context of Type 1 diabetes (T1D) disease progression [84]. It was shown that depletion of Treg in mice expressing the high affinity TCRs resulted in disease acceleration whereas this was not the case for those harboring low affinity Treg. This suggests that low affinity Treg might not be as protective as high affinity Treg. Notably, some of these phenotypes might also be attributable to the frequency of Treg recruited to the inflammation site and/or to the low- versus high-affinity effector cells present in the models. As a follow-up, using models of mixed TCR Tg bone marrow transplantation of high and low self-affinity TCR expressing cells, it was also shown that both low and high affinity Treg had an immunoregulatory function and could suppress T1D development [85]. The authors, however, observed differences in gene expression between low and high affinity Treg, with the latter expressing higher levels of effector molecules such as IL-10, Lag3, CTLA-4 or TIGIT, suggesting that self-reactivity might impart different suppressive mechanisms. This study also suggested that Treg populations with low- and high self-reactivity cooperate through these non-redundant mechanisms to induce protection.

#### 4.1.3. The Strength of Peripheral Stimulation of Treg May Influence Their Suppressive Functions

It is well known that upon thymus egress, nTreg require TCR signaling for their activation into CD44^hi^ effector Treg (eTreg) [86,87,88,89]. This activation has been shown to be dependent on a finely regulated loop involving IRF4 [87,90,91,92,93]. Interestingly, IRF4 transcription factor expression has been shown to be directly proportional to TCR signal strength and acts in a dose-dependent manner on T cell fate [94]. It is thus enticing to speculate that IRF4 levels in Treg, determined by their functional avidity to a peptide could, in part, explain the higher effector function of high affinity Treg.

With the development of TCR Tg cells and mice, the relationship between TCR affinity for antigen and Treg effector function has been further demonstrated. The transduction of human Treg with TCRs of known specificity and affinities to the GAD_555-567_ β-cell peptide, revealed that ex vivo high affinity TCR activation with their cognate peptide augments Treg suppressive ability [95]. Additionally, using a Tg mouse line as a source of monoclonal Treg expressing the B3K506 TCR and APLs, it was shown that low affinity peptides induce a lower immunosuppressive response than their high affinity counterparts which can be rescued by increasing peptide concentration. Overall, the authors show that Treg suppressive function directly correlates with TCR-pMHC avidity (combination of TCR affinity and antigen concentration) [96]. Lastly, work using alloreactive TCR Tg Treg cell lines with different functional avidity to the K^d^ MHC molecule, first demonstrated that high avidity Treg display higher CD69 expression, better suppressive function, and increased in vivo proliferation in a model of allograft rejection [97]. However, using human cord blood Treg transduced with a range of TCRs with well characterized dissociation constants for the SL9 peptide, another group did not detect differences in suppressive activity between these different Treg populations [98].

Overall, it is now well accepted that the strength of TCR signaling both in the thymus and in the periphery dictated by the functional avidity of a T cell for self-peptide is a critical determinant for their differentiation into nTreg and for the establishment of their suppressive phenotype; it is less clear, however, how the strength of TCR signaling received in the periphery influences the phenotype and function of naïve T cells that differentiate into pTreg.

### 4.2. Self-Reactivity Determines the Ability of Naïve CD4^+^ T Cells to Differentiate into pTreg

It is interesting to consider how the strength of TCR signaling perceived in the thymus by naïve conventional CD4^+^ T cells can induce a bias in their ability to differentiate into pTreg following activation. Indeed, several groups have demonstrated that naïve conventional CD4^+^ T cells with relatively high sub-threshold interactions with self-pMHC and expressing high levels of CD5 [84,99] or low levels of Ly6C [80,100] are more efficient at differentiating into pTreg than their CD5^lo^ and Ly6C^hi^ counterparts, respectively. Similarly, in the particular context of Treg depletion in Foxp3-DTR mice, transfer of CD5^hi^ naïve CD4^+^ T cells allows the development of a greater frequency of pTreg than mice injected with CD5^lo^ cells [101]. These data suggest that higher basal TCR interactions with self-pMHC favors Treg induction.

The molecular mechanism(s) responsible for the Treg bias among CD5^hi^ versus Ly6C^-^ CD4^+^ T cells is less well fleshed out and may, in fact, be distinct. The transcriptional profiles of conventional naive CD5^hi^ and Ly6C^-^ CD4^+^ T cells suggest that Treg biases are already present at steady-state prior to Treg induction [23,100]. It has been suggested that differences in calcium flux in Ly6C^-^ versus Ly6C^hi^ cells may account for the predilection of the CD4^+^ T cell population with relatively higher self-reactivity to become Treg [100]. Separately, it has been suggested that relatively higher CD5 levels block the Akt/mTOR mediated inhibition of Foxp3 induction thus promoting Treg differentiation [99,102], while a more recent study suggests that the CD5 signalosome rather restrains the generation of pTreg, through AKT-mediated inhibition of ERK activity [103]. Despite differences in the protocol used for Treg induction in these papers, the cause behind these conflicting observations has yet to be fully understood. The authors of this latest study suggest that the association of the CD5 molecule with different signalosomes (that include, for example, c-CBL or CBL-b) according to the immunological context in which the T cell are activated might explain these striking differences.

### 4.3. pTreg Induction Is Dependent on TCR Signal. Quality

#### 4.3.1. Functional Avidity Is a Critical Component of Foxp3 Induction

Early work on peripheral tolerance suggested that antigen concentration was a major determinant of tolerance induction [104]. In particular, it was observed that two independent mechanisms were at play for the induction of tolerance to orally or intravenously administered antigens. Low doses of antigen resulted in the induction of suppressive CD25 and Foxp3-expressing T cells while higher concentrations of the peptides led to the induction of an anergic state [104,105,106,107,108,109]. It was further shown that sustained delivery of very low doses of antigens, as little as 1 ng/day over 14 days was sufficient to induce these suppressive cells [110]. Since then, this dichotomy between Treg and anergy induction at low and high antigen doses, respectively, has been a staple in the field of oral tolerance.

Later work and the development of different APL have allowed refinement of these initial observations. Using BDC2.5 TCR Tg mice and the low- and high-affinity peptides (KV11 and AV10, respectively), it was demonstrated that low concentrations of high affinity peptide is optimal for the induction of Foxp3-expressing Treg in vitro [111]. Using the 5C.C7 TCR-transgenic model, others further demonstrate that the optimal window of antigen concentration for Foxp3 induction negatively correlates with peptide affinity both in vitro and in vivo [112]. These two seminal studies have hence coined the notion that TCR avidity, integrating both TCR-pMHC affinity and antigen concentration, is a critical parameter of pTreg induction. However, discriminating the relative contribution of these two parameters, affinity and avidity, has proven more difficult. It has been shown, in the context of T cell activation, that peptide affinity is of greater influence than peptide concentration, in terms of sensitivity to IL-2 signaling and that the density of presented peptides can compensate for lower TCR affinities [44,112]. The importance of these pathways for Treg induction has not been fully investigated but suggests that TCR affinity is the main driver of Treg induction, which can be compensated by antigen concentration for lower affinity peptides.

It is important to note that this optimal avidity window for Treg induction is further regulated by cytokines and co-stimulatory molecules. Indeed, cytokines such as TGFβ or retinoic acid stimulation widens this optimal window and allows induction of Treg with a higher concentration of peptide or stronger TCR signaling [112,113]. Additionally, it was shown that CD28 co-stimulation promotes TGFβ-dependent Treg induction at low TCR signal strength but inhibits Treg induction upon stronger TCR signaling [113]. Altogether, the avidity of TCR-pMHC binding, the cytokines and the co-stimulatory molecules define the optimal range of functional avidity for Treg induction in the periphery. Critically, this window of avidity is suboptimal for the proliferation of Foxp3-negative T cells, which is likely to have been evolutionarily selected as a mechanism limiting the activation of autoreactive effector T cell clones having escaped negative selection [111,112].

#### 4.3.2. An Optimal Temporal Window for the Differentiation of pTreg

The integration of signals delivered by an APC over the time of contact is a critical component for thymic T cell selection and T cell activation [70]. In the periphery, TCR signaling duration is also critical for optimal Treg induction. In silico modeling of TCR signaling pathways and their importance in imparting T cell fate revealed that TCR stimulation time is a critical determinant of T cell fate, and further predicted that transient TCR stimulation with a high dose of antigen was optimal for Treg induction [114]. This prediction is backed by experimental demonstration that withdrawal of TCR signaling, either by chemical inhibition of the signaling pathway, removal of the stimuli, or antibody-mediated disruption of the TCR-pMHC interaction results in enhanced Foxp3 induction and Treg frequencies in vitro and in vivo [112,114,115]. Importantly, it has been demonstrated that prolonged TCR signaling by differentiated Treg or constitutive mTOR pathway activation (see below) can antagonize Foxp3 expression both in vitro and in vivo. These results suggest that an optimal temporal window of TCR stimulation, determined by T cell-APC contact time, and participating in overall TCR signaling strength, is required for peripheral induction of Treg.

#### 4.3.3. The Akt/mTOR and the NF-κB Signaling Pathways Integrate TCR Signal Strength for the Induction of Treg

Studies have demonstrated that the PI3K-AKT-mTOR axis of the TCR signaling pathway is a critical negative regulator of Treg fate. Using a constitutively active AKT isoform or chemical inhibitors of PI3K and mTOR, two pioneer studies demonstrated that this signaling pathway negatively regulates Foxp3 induction by naïve T cells [115,116]. Further work demonstrated that S6 phosphorylation, downstream of the Akt signaling cascade, negatively correlates with Treg induction. Lower TCR signaling strength and, hence, lower S6-phophorylation is required for optimal Treg induction. Antigen dose rather than TCR affinity appears to be the major factor in the encoding of the PI3K-Akt-mTOR signal, however a more detailed study considering functional avidity as a whole for controlling this pathway is lacking [111,117]. Mechanistically, it was further demonstrated that TCR signal strength led to the generation of different phosphatidylinositols, regulated Akt phosphorylation status and its substrate specificity [117,118]. Interestingly, it has also been proposed that an NF-κB-dependent mechanism, involving IFNγ and TNF, among other cytokines, also suppresses the induction of Foxp3 at high doses of TCR stimulation [109]. The authors suggest that high doses of TCR stimulation promote the differentiation of T cells to proinflammatory subsets despite the presence of a Treg differentiating milieu.

### 4.4. TCR Signaling and Treg Persistence in the Periphery

Given the higher basal TCR interactions with self-pMHC of Treg as compared to naïve CD4^+^ T cells [69], it was hypothesized that this tonic TCR signaling was required for their long-term maintenance in the periphery. However, several studies have demonstrated that TCR signaling is not required for the maintenance of Foxp3 expression in the periphery. Despite the loss of their immunosuppressive function, TCR deficient Treg are maintained up to several weeks after TCR ablation [86,87,88]. However, it is interesting to note that these Treg have a much lower proliferation rate than their TCR competent counterparts, and thus a slightly higher decay rate and a higher propensity to undergo apoptosis [87,88]. It was also reported that TCR-deficient Treg also have an altered distribution in the periphery [86].

The importance of TCR signaling strength for the maintenance of Treg in the periphery has been poorly investigated. However, it has been demonstrated that Treg induced by weak agonist peptide persist for a shorter amount of time than Treg induced by high-affinity ligand [112]. While this study did not investigate the relative contribution of TCR signaling strength, antigen persistence and competition for the antigen with Foxp3^-^ T cells, it suggests that an optimal TCR signaling strength is required for long-term maintenance of functional Treg.

### 4.5. TCR Signaling Shapes Treg Heterogeneity: Considerations and Open Questions

It is important to note that all these studies display a wide diversity in the (1) methods used to measure suppressive function (in vitro inhibition of proliferation vs in vivo measures of disease progression), (2) methods for measuring TCR affinity (functional measures, surface plasmon resonance, 2D micropipette adhesion), and (3) methods of Treg stimulation (peptide stimulation vs APC), which are likely to explain seemingly contradictory conclusions. It is also important to highlight the limits of using monoclonal TCR Tg T cell population which involves significant intraclonal competition and might not fully reflect the variability of TCR affinity in the polyclonal population.

Despite these caveats, the quality of the TCR signals that accompany nTreg development in the thymus clearly influences Treg function. In addition, the quality of TCR signals that accompany ‘activation’ of differentiated Treg in the periphery appears to impact the quality of their immunoregulatory activity; though some contradictory observations prevent a full conclusion relevant to the role of TCR avidity on the suppressive function of monoclonal Treg populations. What is less clear is the influence of TCR signals during pTreg induction on their suppressive abilities. Altogether, the studies presented suggest that the induction of pTreg from naïve T cells in the periphery requires a ‘sweet spot’ of TCR signaling. Modulation of TCR-pMHC affinity, pMHC density, levels of co-stimulation and cytokine composition of the milieu, and TCR signaling time determines TCR signaling strength and the efficiency of pTreg induction; whether and how these individual factors influence the ‘quality’ of pTreg that develop requires additional study (Figure 2).

## 5. Clinical Perspectives

Consideration of heterogeneity among anergic and Treg cell populations and how the strength of TCR signal induces functional biases among individual cells in these subsets has important clinical implications, particularly in the context of emerging immunotherapies. Many of these immune cell therapies act via delivery of a bolus of T cells to modulate the immune response or through the mobilization of endogenous T cells. Consideration of tolerant T cell phenotypes is critical in terms of improving the efficacy of their therapeutic potential as well as limiting adverse side-effects.

Adoptive T cell therapies rely on the ex vivo expansion or differentiation of T cells from a polyclonal T cell population or from a genetically modified population expressing specific TCR or chimeric antigen receptors [119]. Anti-tumor T cell therapies have demonstrated particularly promising results in the treatment of blood cancers [120]. Treg therapies, or Treg ‘enhancing’ approaches, are at a more immature stage of development, but have met with great enthusiasm for the suppression of immune responses in the context of autoimmune diseases, transplant rejection, and graft-versus-host disease [121,122,123]. Thus, the ability to better understand how the fine tuning of TCR signal strength can influence Treg induction and function is of great importance. In particular, relatively stronger TCR signals in the thymus appear to drive production of more suppressive Treg cells. Thus, the quality of TCR signals provided may be an important consideration for improving the efficacy of an in vitro differentiated Treg product. At the same time, indicators of the TCR signal strength that accompany Treg differentiation (e.g., CD5) may allow the parsing of Treg populations with the most appropriate level of immunoregulatory function [28,29,30].

On the other hand, checkpoint blockade immunotherapies target co-signaling molecules that belong to the immunoglobulin and tumor necrosis factor receptor superfamilies [124]. These co-signaling molecules positively or negatively regulate the T cell response. Tumors can evade T cell killing by up-regulating co-inhibitory proteins or recruiting immunosuppressive cells expressing negative regulators of T cell activity; thus, co-signaling proteins have become important targets of cancer immunotherapy [125,126]. Indeed, when used as single or combinatorial agents, antibodies blocking CTLA-4, PD-1, PD-L1, and other checkpoint molecules activate anti-tumor T cells and have had success in the clinic for the treatment of skin and lung cancers among others, significantly extending the lives of patients [126,127,128,129,130]. However, severe immune toxicities have been observed in a significant frequency of treated subjects [131]. These immune-related adverse events (irAEs) often limit continuation of checkpoint blockade therapies and may cause lifelong morbidities. For example, checkpoint blockade can lead to de novo insulin-dependent diabetes; though to what extent checkpoint inhibitor induced irAEs mimic their autoimmune disease counterparts is less clear [132,133]. These observations emphasize the important balance between T cell activation and tolerance, as well as our need to understand the specific subsets of cells re-activated by checkpoint inhibitors. For example, anti-PD-1 treatments have been shown to reverse tolerance allowing for cells to be activated and cause immune pathologies [26]. An elegant study has also shown that tolerant T cells are able to move freely near DC expressing cognate peptide within lymph nodes. The motility of these cells was decreased following blockade of PD-1 pathways, allowing tolerant T cells to engage with DC, which in turn led to the development of immune pathologies [134]. On the other hand, it is important to note that some tolerant T cells are resistant to anti-PD-1 blocking therapy [27]. Nevertheless, tolerance can be reversed following strong TCR signaling in the presence of inflammatory cues, which renders these cells susceptible to blocking therapies [59]. It is tempting to speculate that heterogeneity in sensitivity to anti-PD-1 exists among anergic T cell populations and that this is imprinted based on the strength of TCR signal induced during anergy induction. Indeed, accumulating evidence implicates rather low affinity T cells in the development of autoimmune pathologies illustrating the importance of considering the entire spectrum of TCR affinity to self in terms of the efficiency and ‘quality’ of tolerance induction [4,22,52,135].

## 6. Conclusions

The influence of TCR signal strength in the determination of T cell fate is increasingly appreciated as an important factor in the field of T cell tolerance. In the context of peripheral tolerance to self and self-associated antigens, we highlighted the importance of TCR affinity, avidity, and functional avidity in driving the diversity of anergic and regulatory T cell fate and function. There is clear evidence that differences in the affinity/avidity of TCR interactions with pMHC during tolerance induction in either the thymus or periphery lead to differences in TCR signal propagation, Ca^2+^ flux, gene expression, and, in some cases, fate determination. Although the precise mechanism is yet to be fully uncovered, the strength of TCR signaling is known to play a role in the establishment of CD4^+^ and CD8^+^ T cell anergy; despite this, the induction of anergy in autoreactive T cells occurs over a wide range of functional avidity to self [52,53,54,65]. Subtle changes in the strength of TCR signals perceived by T cells under tolerizing conditions may explain the heterogeneity observed in anergic T cell states though this has yet to be formally tested [65]. The role of TCR signal strength in Treg heterogeneity is a bit clearer, at least during nTreg induction. The strength of TCR interactions with self-peptide in the thymus lead to distinct Treg progenitors which, ultimately, generate nTreg with differences in their suppressive functions [28,29]. Whether this is also true for the induction of pTreg from naïve T cells following recognition of self- or self-associated peptides is yet to be definitively determined.

Overall, these studies highlight the importance of functional avidity to self in driving the diversity of tolerant phenotypes in the periphery. What is lacking is comprehensive analyses of the levels of self-reactivity that induce tolerant T cells relative to polyclonal conventional T cell populations that undergo positive and negative selection as well as a link between affinity/avidity, TCR signals, and anergic T cell or pTreg phenotype. We believe that a systematic dissection of the specific involvement of TCR affinity, avidity, and functional avidity through the rigorous use of APL, TCR Tg mice or variation of peptide concentration, and with the knowledge of the caveats associated with these techniques, is critically required. Additional layers of complexity, such as the heterogeneity of the peripheral compartments in which tolerance is induced and sex-biases, among other factors, will need to be further accounted for in such studies.

Different subpopulations of anergic T cells and Treg likely have complementary, non-redundant roles in maintaining self-tolerance. Thus, further understanding of the impact of TCR signal strength in imprinting the phenotypes in these tolerant T cell states is of great interest. These studies are critical not only for understanding how tolerance to self is maintained but also to develop new or improved approaches for the control and treatment of autoimmune manifestations.
